#  Generation of low-flux X-ray micro-planar beams and their biological effect on a murine subcutaneous tumor model

**DOI:** 10.1093/jrr/rrv037

**Published:** 2015-07-03

**Authors:** Zhengshan Hong, Junko Zenkoh, Biao Le, Ariungerel Gerelchuluun, Kenshi Suzuki, Takashi Moritake, Masakazu Washio, Junji Urakawa, Koji Tsuboi

**Affiliations:** 1 Proton Medical Research Center, Faculty of Medicine, University of Tsukuba, 1-1-1 Tennodai, Tsukuba, Ibaraki 305-8575, Japan; 2 Ningbo M&J Biotechnologies Co. Ltd, 299 GuangHua Road, Building C6, Suite 1, High-tech Zone, Ningbo City, China; 3 Department of Radiological Health Science, Institute of Industrial Ecological Sciences, University of Occupational and Environmental Health, 1-1 Iseigaoka, Yahatanishi-ku, Kitakyushu, Fukuoka 807-8555, Japan; 4 Research Institute for Science and Engineering, Waseda University, 3-4-1 Okubo, Shinjuku-ku, Tokyo 169-8555, Japan; 5 High Energy Accelerator Research Organization, 1-1 Oho, Tsukuba, Ibaraki 305-0801, Japan

**Keywords:** micro-planar beams, antitumor effects, skin reaction, bystander effect

## Abstract

We generated low-flux X-ray micro-planar beams (MPBs) using a laboratory-scale industrial X-ray generator (60 kV/20 mA) with custom-made collimators with three different peak/pitch widths (50/200 μm, 100/400 μm, 50/400 μm). To evaluate normal skin reactions, the thighs of C3H/HeN mice were exposed to 100 and 200 Gy MPBs in comparison with broad beams (20, 30, 40, 50, 60 Gy). Antitumor effects of MPBs were evaluated in C3H/HeN mice with subcutaneous tumors (SCCVII). After the tumors were irradiated with 100 and 200 Gy MPBs and 20 and 30 Gy broad beams, the tumor sizes were measured and survival analyses were performed. In addition, the tumors were excised and immunohistochemically examined to detect γ-H2AX, ki67 and CD34. It was shown that antitumor effects of 200 Gy MPBs at 50/200 μm and 100/400 μm were significantly greater than those of 20 Gy broad beams, and were comparable with 30 Gy broad beams. γ-H2AX-positive cells demonstrated clear stripe-patterns after MPB irradiation; the pattern gradually faded and intermixed over 24 h. The chronological changes in ki67 positivity did not differ between MPBs and broad beams, whereas the CD34-positive area decreased significantly more in MPBs than in broad beams. In addition, it was shown that skin injury after MPB irradiation was significantly milder when compared with broad-beam irradiation at equivalent doses for achieving the same tumor control effect. Bystander effect and tumor vessel injury may be the mechanism contributing to the efficacy of MPBs.

## INTRODUCTION

The application of micro-planar beams (MPBs) to radiotherapy for solid cancers has been explored because they have the potential to target tumor tissues with less normal tissue toxicity compared with conventional broad beams. The original report was by Baker *et al.* in 1961 [1], in which an apparatus that generated 25-μm deuteron beams was developed for biological study. Simulating cosmic particle-beam irradiation, mice were irradiated with 25 µm 22-MeV deuteron beams with unexpectedly minimal somatic damage [2]. Based on this observation, Slatkin *et al.* of Brookhaven National Laboratory used synchrotron-generated high-flux X-rays (50–150 keV) to generate MPBs that were irradiated to the heads of rats, reporting an absence of brain necrosis even at the skin-entrance doses of 312–5000 Gy [3]. In 1998, similar synchrotron-generated MPBs were used for rat brain tumor models, in which 9L gliosarcomas were implanted in syngeneic rat brains, to examine the therapeutic efficacy [4]. MPBs used in their study were as follows: center-to-center distances of 100 µm, slice widths of 25 µm, peak doses of 312.5 Gy, and total doses of 625 Gy. The results demonstrated that the median survival times of tumor-bearing rats treated with these MPBs were significantly longer than those of the untreated control group, and the toxicity was within a tolerable range [4].

Following these reports, MPB-based experiments were also initiated using the synchrotron at the European Synchrotron Radiation Facility, Grenoble, France and at SPring-8 in Hyogo, Japan. They developed a custom-made multi-slit collimator and confirmed that generated MPBs were applicable for basic animal experiments with enough contrast between the peak and valley doses [5]. The 9L rat brain tumor model [6, 7] or mouse hind limb models [8, 9] were used to confirm the efficacy of MPB irradiation in these facilities.

So far, it has been demonstrated that a single fraction of 100 Gy of synchrotron-generated X-ray MPBs had the potential to target cancer tissues while preserving normal tissues fairly well. However, MPBs have not been clinically applied yet, partly because of the insufficiency of biological data. This may be due to the fact that only synchrotrons have been used to generate high-flux X-rays. Since not all institutes have free access to synchrotrons, the construction of a more compact and accessible system would possibly contribute to the accumulation of biological data, eventually leading to clinical application. Thus, in this study, we tried to develop a system to apply low-flux X-ray MPBs generated by a laboratory-scale industrial X-ray generator. We then explored whether the same biological effects could be obtained using our low-flux MPB system. The results presented here should help us to understand the biological effects of MPBs, and facilitate preclinical research on MPB-based radiotherapy.

## MATERIALS AND METHODS

### Ethics

The animal experimental procedures were submitted to the University of Tsukuba Animal Experiment Committee and approved on 11 May 2012.

### Experimental setup and dosimetry of X-ray broad beams and micro-planar beams

**Fig. 1.**
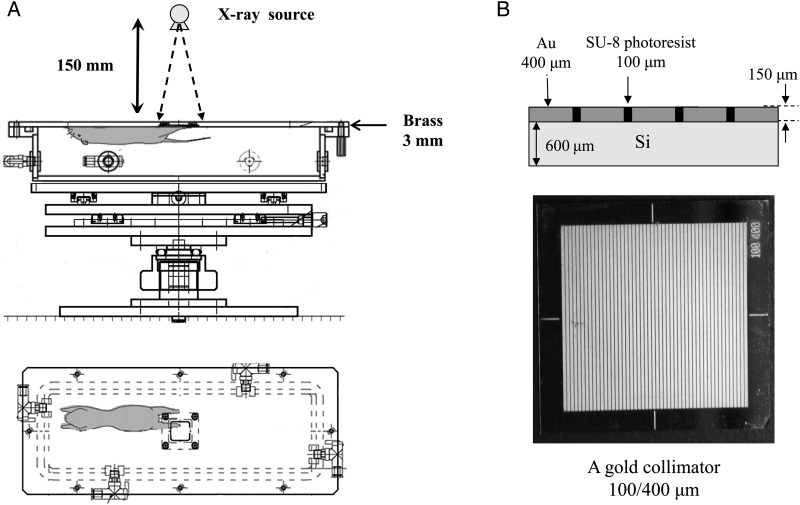
(**A**) An original container for irradiation of the mouse left hind limb through a window measuring 16 mm × 16 mm, to which the gold grid collimators on the silicon substrate or the silicon substrate alone were mounted. This container was set inside the laboratory-scale X-ray generator. (**B**) A gold grid collimator made of 150-µm-thick stripe-patterned gold fabricated on the 600-µm-thick silicon substrates. The widths of the SU-8 photoresist/gold were set at either 50/200 µm, 100/400 µm or 50/400 µm. One presented here is 100/400 µm.

We constructed an original container for the irradiation of the mouse left hind limb (Fig. 1A). The top of the container was made of 3-mm-thick brass with a window measuring 16 mm × 16 mm, to which the gold grid collimators (Fig. 1B) on the silicon substrate or the silicon substrate alone were mounted.

Both conventional broad beams and MPBs were generated using an X-ray generator MBR-1520R (Hitachi Medical Co., Tokyo, Japan) operating at 60 kV and 20 mA, with a 1.0-mm aluminum filter at the Laboratory Animal Resource Center, the University of Tsukuba.

To generate the stripe-pattern X-ray MPBs, we used three gold grid collimators (Fig. 1B) made of 150-µm thick stripe-patterned gold fabricated on the 600-µm-thick silicon substrates. The widths of the SU-8 photoresist/gold were set at 50/200 µm, 100/400 µm or 50/400 µm (Fig. 1B).

**Fig. 2.**
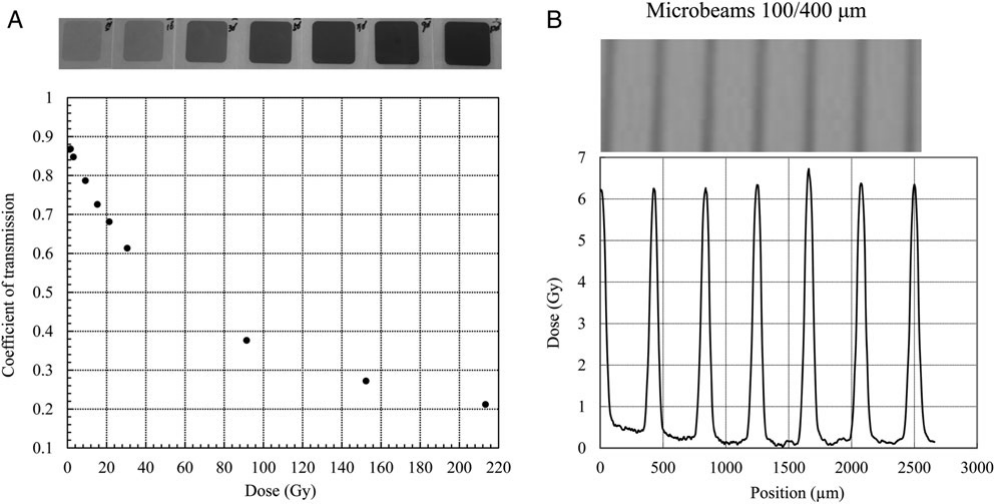
(**A**) The GafChromic films attached behind the glass dosimeter were exposed to broad-beam X-rays through the container window at different doses, and the film transmittance was measured to draw the transmittance-to-dose response curve. (**B**) Using this curve, the peak and valley doses of MPBs were calculated after the films were exposed through the gold grid collimators. The peak-to-valley dose ratio of MPBs of 100/400 µm presented here was ∼10:1.

To measure the absorbed dose of the broad beams, we used a glass dosimeter (ACG Techno Glass Co. Ltd, Shizuoka, Japan) fixed at the center of the window of the brass plate of the container. The absorbed dose was evaluated by a dosimeter reader (FGD-1000 Chiyoda Technol Corp, Tokyo, Japan). We used GafChromic films (ISP Technologies Inc., Waterford, Michigan, USA) for the dosimetry of MPBs (Fig. 2). First, the GafChromic films attached behind the glass dosimeter were exposed to broad-beam X-rays through the container window at a range of doses, and the film transmittance was measured using a microdensitometer to draw the transmittance-to-dose response curve (Fig. 2A). Using this curve, we then calculated the peak and valley doses of MPBs after the films were exposed through the gold grid collimators (Fig. 2B). This revealed peak MPB dose rates of 1.94 Gy/min, 1.86 Gy/min and 2.28 Gy/min with 50/400-µm, 50/200-µm and 100/400-µm beams, respectively. The peak-to-valley dose ratio was estimated to be >10.

### Skin reactions induced by MPB and broad-beam irradiation

One week before the irradiation, the left legs and thighs of C3H/HeN mice (Clea Japan Inc., Tokyo, Japan) were chemically depilated and thoroughly soaked with saline. The mice were then anesthetized with sodium pentobarbital in saline (50 mg/kg i.p.) (Tokyo Chemical Industry Co. Ltd., Tokyo, Japan) and immobilized against the container using adhesive tape (Fig. 1). For the conventional broad-beam irradiation, single doses of 20, 30, 40, 50 and 60 Gy were selected based on the report of Iwakawa *et al.* [10]. As for MPBs, single fractions were delivered with peak doses of 100 or 200 Gy using the three collimators (50/400 µm, 50/200 µm and 100/400 µm). MPB doses were determined based on the report of Griffin *et al.* [11], in which peak doses of 75 and 150 Gy were given. In addition, the maximum irradiation time was dependent on the anesthetic effect that persisted for ∼100 min and corresponded to a 200-Gy peak dose with our X-ray generator. Each group comprised five mice. The acute skin reaction after irradiation was scored every 2–3 days for 60 days using the arbitrary scale [10, 12].

### Antitumor effects induced by MPB and broad-beam irradiation

Murine squamous cell carcinoma (SCCVII) cells were kindly donated by Dr Koichi Ando of Gunma University, Japan. The cells were grown in minimum essential medium (MEM) (Sigma–Aldrich Inc., Tokyo, Japan) supplemented with 100 µg/ml streptomycin, 100 U/ml penicillin (Sigma–Aldrich Inc.) and 10% fetal bovine serum (FBS) (Sigma–Aldrich Inc.). Then, 1 × 10^5^ SCCVII cells suspended in 100 µl MEM without FBS and antibiotics were subcutaneously implanted into the left thighs of the C3H/HeN mice. Approximately 7 days after inoculation, the tumor diameters were 5–6 mm, as measured by calipers.

The tumor-bearing mice were anesthetized and fixed to the container as described above, and the thigh tumors were exposed to single fractions of broad beams at 20 and 30 Gy, or to three types of MPBs at 100 and 200 Gy peak doses. Each group comprised five mice. The tumor volumes were measured every 2–3 days using a caliper until the tumor size was ∼20 mm × 20 mm according to our animal experiment protocol in compliance with the ‘Guideline for Endpoints in Animal Study Proposals’ of the NIH [13]. At this point, the mice were sacrificed by cervical dislocation.

### Immunohistochemical analyses

After being irradiated with 200 Gy peak doses of 50/200 µm and 100/400 µm MPBs and 30 Gy of conventional broad beams, the tumors were excised from the mice immediately after cervical dislocation at 1 h, 3 h, 9 h, 1 day, 4 days and 7 days. The tumor tissues were fixed in 10% formalin for 24 h prior to paraffin embedding. On glass slides, the specimens were dewaxed (using xylene) and boiled in a 10-mM sodium citrate buffer (pH 6.0) for 20 min, and then slowly cooled to room temperature. Furthermore, the sections were incubated in a 3% hydrogen peroxide solution for 5 min and washed in phosphate-buffered saline (PBS) twice. The treated specimens on the slides were immunostained to detect a DNA double-strand break marker (γ-H2AX) [14], a cell proliferation marker (ki67) [15] and an endothelial marker (CD34) [16].

For γ-H2AX staining, specimens were blocked by M.O.M.™ mouse Ig-blocking reagent (Vector Laboratories Inc., California, USA) for 60 min at room temperature. Then, they were incubated with an anti-phospho-histone H2A.X (Ser139) antibody (Millipore Corporation, Billerica, MA, USA) diluted with Can Get Signal® immunoreaction enhancer solution-A (Toyobo Ltd, Osaka, Japan) for 60 min at room temperature. After washing with PBS twice, the specimens were incubated in M.O.M.™ biotinylated anti-mouse immunoglobulin G (IgG) antibody (Vector Laboratories Inc., California, USA), diluted with Can Get Signal® immunoreaction enhancer solution-A for 60 min at room temperature, and washed in PBS twice. Finally, the specimens were stained by biotin-streptavidin using the LSAB®2 system-HRP (DAKO, California, USA) following the manufacturer's instructions. When the specimens became brown in color the reaction was stopped by rinsing with PBS. The specimens were dehydrated, penetrated, and covered by glass on slides.

For the CD34 staining, the specimens were blocked on the slides with the blocking buffer of the BupH™ Tris-Buffered Saline Pack (Thermo Fisher Scientific Inc., Waltham, USA) for 30 min at room temperature. Then, they were incubated with an anti-CD34 monoclonal antibody (Hycult Biotech, Uden, Netherlands) diluted by the blocking buffer of peroxidase detection reagent Pack (Thermo Fisher Scientific) for 2 h at 37°C and washed with PBS twice. The specimens were incubated in biotinylated rabbit anti-rat IgG (DAKO) diluted by the blocking buffer (Thermo Fisher Scientific) for 60 min at room temperature and washed with PBS twice. Finally, the specimens were stained by biotin-streptavidin using the LSAB®2 system-HRP (DAKO) according to the manufacturer's instructions. When the specimens became brown in color the reaction was stopped by rinsing with PBS. The specimens were dehydrated, penetrated, and covered by glass on the slides. Images were captured by the microscope (Biozero BZ-8000 KEYENCE; Tokyo, Japan), and three uniformly stained fields were selected. The CD34-positive areas were calculated by image-J software (National Institutes of Health, Bethesda, USA).

For ki67 staining, specimens were blocked with 10% goat serum (Vector Laboratories Inc., Burlingame, USA) for 60 min at room temperature. Then, they were incubated with an anti-ki67 antibody (Abcam Inc., Cambridge, UK) diluted by 1% goat serum for 60 min at room temperature and washed with PBS twice. Finally, the specimens were stained by biotin-streptavidin using the LSAB®2 system-HRP (DAKO) following the manufacturer's instructions. When the specimens became brown in color the reaction was stopped by rinsing with PBS. The specimens on the slides were dehydrated, penetrated, and covered by glass. The positivity of ki67 was calculated as the percentage of positive cells among 500 cells counted in three uniformly stained fields under the microscope (Biozero BZ-8000).

We used two sets of 12 tumors (a total of 24 tumors) for immunohistochemical analyses of γ-H2AX at 1, 3, 9 and 24 h after 30 Gy broad-beam and 200 Gy of both 50/200 μm and 100/400 μm MPB irradiation. For both CD34 and Ki67 positivity evaluation, three fields from one section of two mice, a total of six fields, were used for calculation of *P*-values.

## RESULTS

### Acute skin reactions

**Fig. 3.**
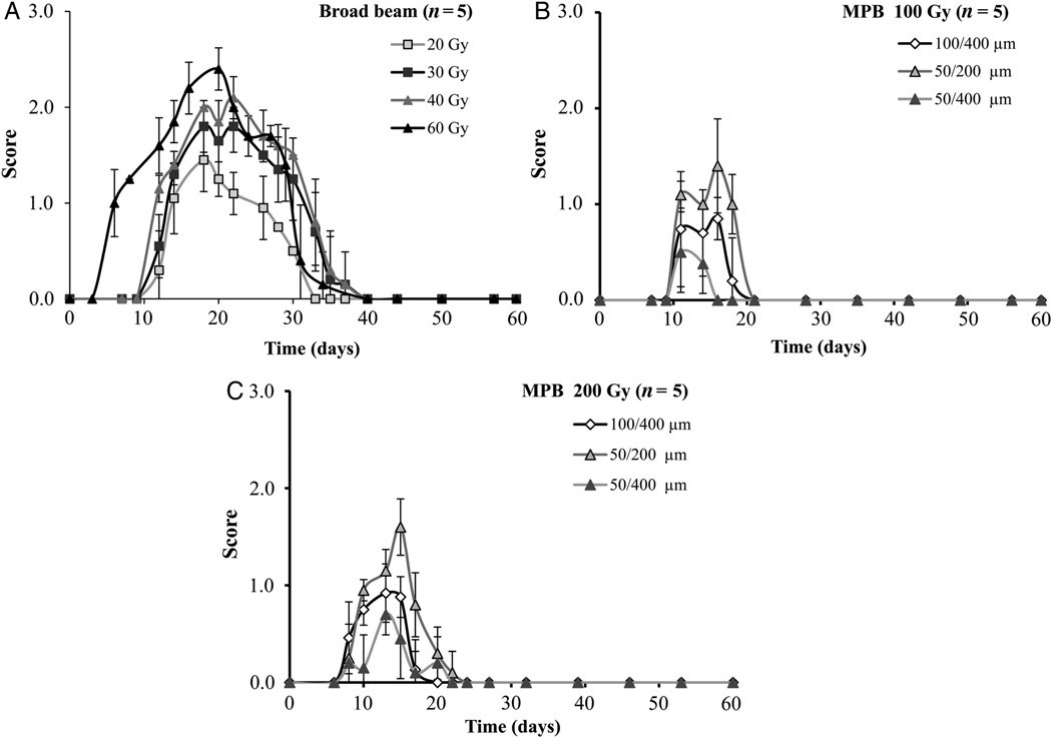
Skin reactions induced by broad beam (**A**) or MPB (**B, C**) irradiation. The horizontal bars indicate date, and the vertical bars indicate the acute skin reaction score. (**A**) Skin reaction after broad beam irradiation. (**B**) Skin reaction after 100 Gy MPB irradiation. (**C**) Skin reaction after 200 Gy MPB irradiation.

After broad-beam irradiation at doses of 20–60 Gy, acute dermatitis appeared in 7–10 days in a dose-dependent manner. The peak occurrence of dermatitis was at Day 20, with scores of 2.4, 2.1, 2.1, 1.8 and 1.5 for 60, 50, 40, 30 and 20 Gy, respectively. However, the damage spontaneously healed by Days 35–40 (Fig. 3A). In contrast, acute dermatitis appeared by approximately Day 9 after the three types of MPB irradiation at 100 Gy (Fig. 3B). The dermatitis peaked between Days 11 and 16, with scores of 1.4, 0.9 and 0.4 for 50/200 µm, 100/400 µm and 50/400 µm, respectively. It appeared by Day 8 for the 200 Gy dose and peaked between Days 13 and 15, with scores of 1.6, 0.9 and 0.7 for 50/200 µm, 100/400 µm and 50/400 µm, respectively (Fig. 3C). The acute dermatitis after MPB irradiation was repaired within 22 days for the 100 Gy dose and within 24 days for the 200 Gy dose (Fig. 3B and C). When comparing the 200 Gy MPB irradiation (50/200 µm, 100/400 µm and 50/400 µm) with the 20 and 30 Gy conventional broad-beam irradiation, repair of the acute skin reaction was significantly faster for MPBs. The peak dermatitis score for the 200 Gy 50/200 µm MPB irradiation was almost equivalent to the 20 Gy broad-beam irradiation and lower than that for the 30-Gy broad-beam irradiation. In addition, the peak scores were lower for both the 200 Gy 50/400 µm and 200 Gy 100/400 µm irradiations than they were for the 20 Gy broad-beam irradiation.

### Tumor growth delay and survival analysis *in vivo*

**Fig. 4.**
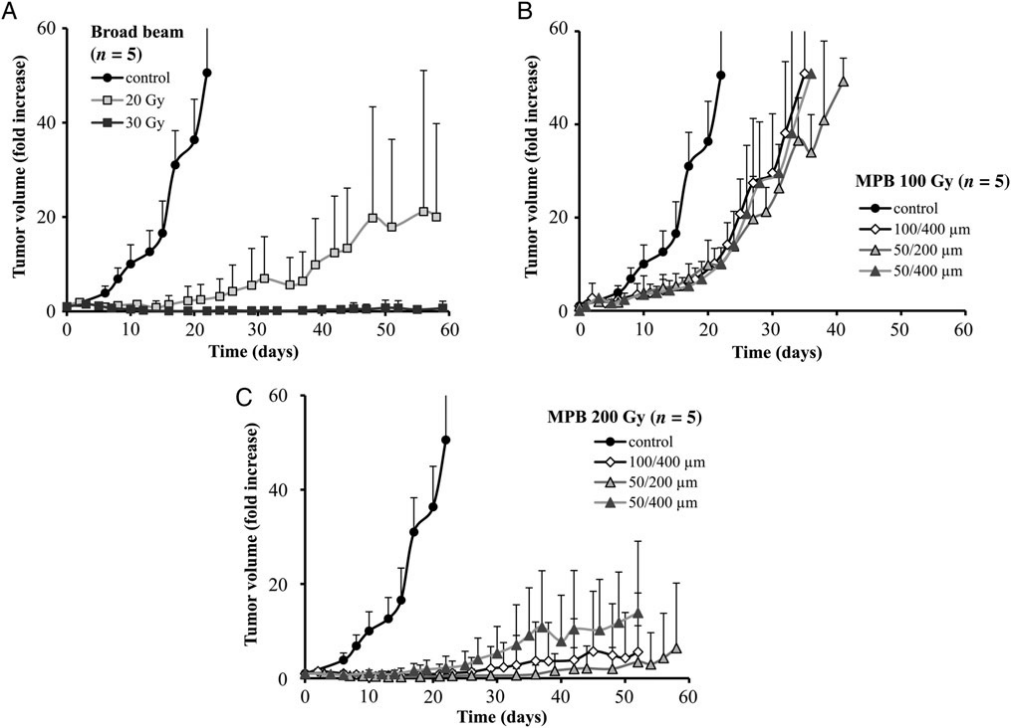
Tumor growth curves after irradiation with broad beams (**A**) or MPBs (**B, C**). The horizontal bars indicate date, and the vertical bars indicate the tumor volumes presented in fold-increase. (**A**) Tumor growth curves after irradiation with broad beams (20, 30 Gy). (**B**) Tumor growth curves after irradiation with three kinds (100/400 µm, 50/200 µm, 50/400 µm) of 100-Gy MPBs. (**C**) Tumor growth curves after irradiation with three kinds (100/400 µm, 50/200 µm, 50/400 µm) of 200-Gy MPBs.

The growth of the subcutaneously implanted SCCVII tumor was suppressed following broad-beam irradiation in a dose-dependent manner. Single fraction 30 Gy broad beams suppressed tumor growth during the 60-day observation period (Fig. 4A). When irradiated with a 100 Gy MPB peak dose, tumor growth was delayed but did not significantly differ between the three MPBs (50/200 µm, 100/400 µm and 50/400 µm) (Fig. 4B). In contrast, 200 Gy MPB peak doses suppressed the tumor growth more efficiently than did 100 Gy doses. Both 50/200 µm and 100/400 µm were more effective than 50/400 µm (Fig. 4C). When comparing 200 Gy MPBs (50/200 µm, 100/400 µm and 50/400 µm) against 20 and 30 Gy broad beams, the tumor growth suppression effect occurred in the following order: 30 Gy ≥ 50/200 µm ≥ 100/400 µm > 50/400 µm > 20 Gy.

**Fig. 5.**
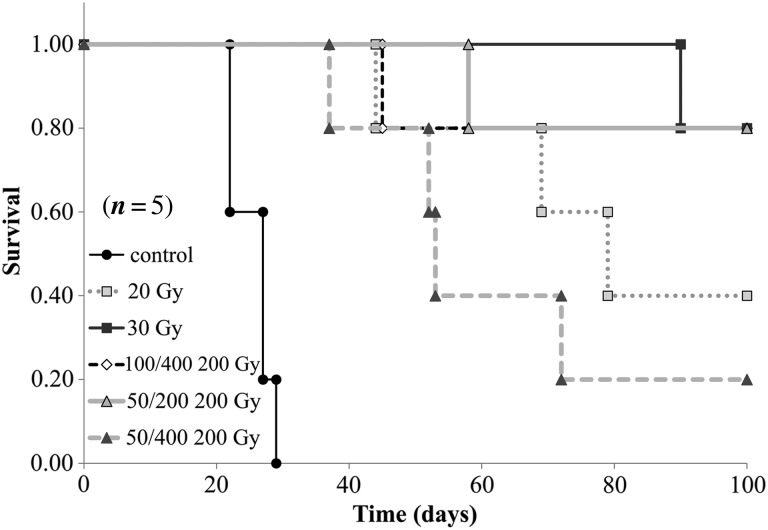
The Kaplan–Meier survival analysis. The horizontal bars indicate date, and the vertical bars indicate survival ratio.

When the tumor size reached ∼20 × 20 mm, mice were sacrificed by cervical dislocation, and survival analysis was performed by the Kaplan–Meier method. The resulting survival curves demonstrated that differences between the 30 Gy broad beams and the 200 Gy 50/200 µm and 100/400 µm MPBs were insignificant, with *P*-values of 0.94 and 0.52, respectively. In contrast, 200 Gy 50/400 µm MPB irradiation had significantly lower effects than the 30 Gy broad beams (*P* = 0.037) (Fig. 5).

### Immunohistochemical analyses

**Fig. 6.**
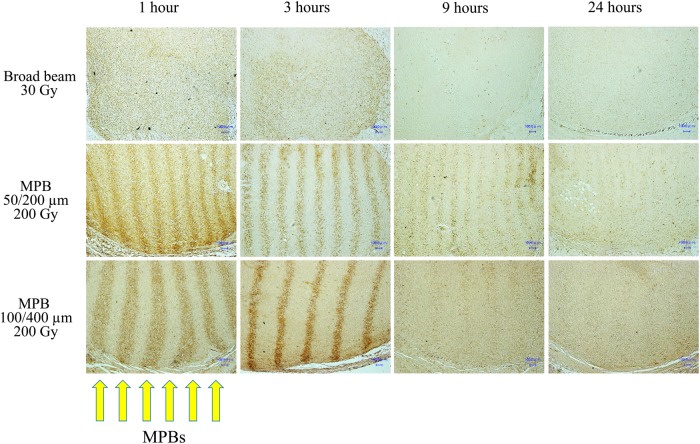
Microphotographs of tumor tissues immunohistochemically stained by anti-γ-H2AX monoclonal antibody.

The γ-H2AX-positive cells demonstrated clear stripe patterns 1 and 3 h after irradiation with 200 Gy of both the 50/200 µm and 100/400 µm MPBs (Fig. 6). The stripes gradually faded by 9 h and had almost disappeared by 24 h. In particular, the bands made by MPB of 100/400 μm became dense and narrow at 3 h, then they became wider and faded in 9 and 24 h, respectively, probably indicating intermixing of positive and negative cells. In addition, the stripe pattern was observed throughout the tumor sections examined, indicating that MPBs reached a depth of at least 1 cm from the skin surface.

**Fig. 7.**
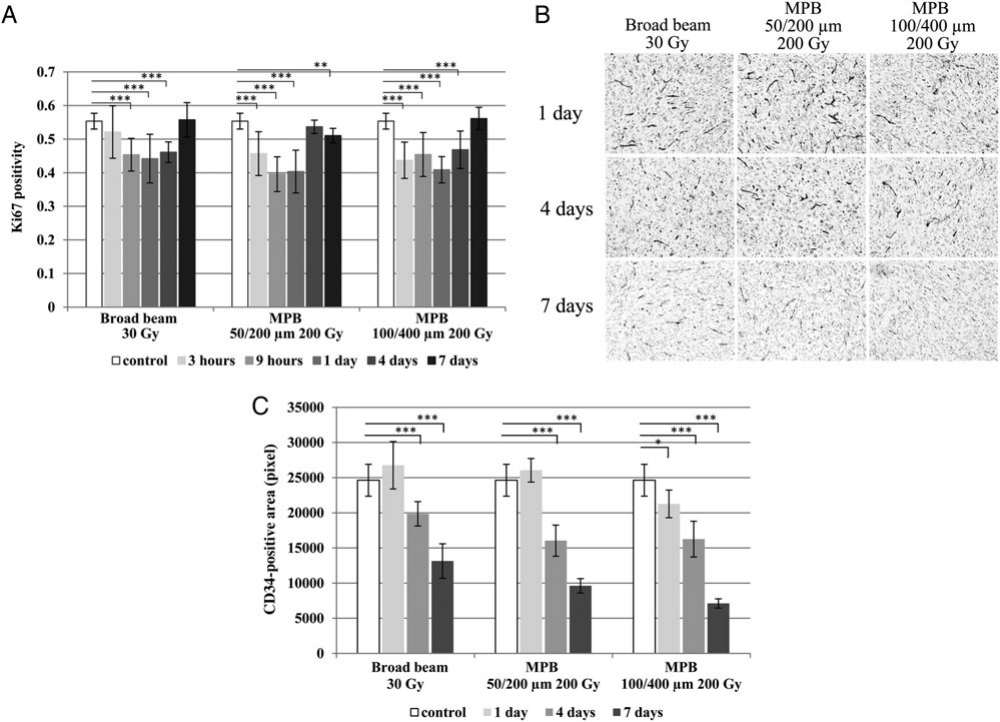
(**A**) Serial changes of ki67 positivity until 7 days after each irradiation (**P < 0.01, ***P < 0.005). (**B**) Microphotographs of tumor tissues immunohistochemically stained by anti-CD34 monoclonal antibody. (**C**) Serial changes of CD34-positive area until 7 days after each irradiation (*P < 0.05, ***P < 0.005).

The ki67 positivity decreased with time after the irradiation, until 9 h or 1 day, and then gradually returned to the control level by Day 7. This pattern was similar for the 30 Gy broad beams and the 50/200 µm and 100/400 µm MPBs. Thus, at equivalent doses for *in vivo* tumor growth inhibition, the number of tumor cycling cells had similar chronological patterns after irradiation with either broad beams or MPBs (Fig. 7A).

CD34-positive areas were significantly reduced by 4–7 days for all irradiation types when they were compared with the non-irradiated controls. In particular, the CD34-positive area was significantly decreased by the end of Day 1 for the 100/400 µm MPB; the CD34-positive area for the 100/400 µm MPB was also significantly smaller than was observed for the 30-Gy broad-beams (*P* = 0.0003) or the 50/200 µm MPB (*P* = 0.0009) by Day 7 (Fig. 7B and C).

## DISCUSSION

It has been reported that synchrotron-generated X-ray MPBs could preferentially kill cancer cells while being less toxic to normal tissue [17, 18]. Based on this potential advantage, the possibility for clinical application has been explored. However, it is necessary to clarify the biological effects of MPB irradiation and to collect reproducible biological evidence of their efficacy first if it is to be accepted as a new modality of cancer radiotherapy. The merit of radiotherapy using MPBs that intentionally leaves cancer tissue and normal tissue in a micro order grid pattern has not been fully investigated by current radiation oncology, in which the aim is for tumor tissues to be completely covered by the irradiation field. Thus, in this study, we tried to develop a useful system for generating low-flux X-ray MPBs without using synchrotrons, and tried to clarify whether our low-flux MPBs could induce similar favorable biological effects to those reported for synchrotron-generated high-flux X-rays.

### Effect of X-ray sources

Synchrotron-generated high-flux X-rays have been used to generate MPBs for animal experiments. The main advantages of synchrotron sources are their extremely high-dose rate and small divergence of beams. In the SPring-8 synchrotron (Hyogo, Japan), peaks of 137 Gy/s [19] or 124 or 111 Gy/s [20] were used, depending on the width of MPBs. The dose rates at the peaks of MPBs in our study were 0.032 Gy/s with 50/400 µm beams, 0.031 Gy/s with 50/200 µm beams, and 0.038 Gy/s with 100/400 µm beams, which is ∼1/3600th of those used by SPring-8. Although the biological effect is expected to be higher with this extremely high dose rate, there have been no precise data comparing the hundreds dose/s and 2 Gy/min to our knowledge.

In our model, in which SCCVII cells were subcutaneously implanted into the left thigh of C3H/HeN mice, we observed similar patterns of delay in tumor growth after MPB irradiation to those reported by Griffin *et al.*, in which mouse mammary tumor cells (4T1) were implanted into the right hind limbs of BALB/c mice. For example, delay in tumor growth after irradiation with 200 Gy of 50/200 µm in our study (Fig. 4) was at least comparable with that after irradiation with 150 Gy of 50/200 µm in their data [11]. In addition, our immunohistochemistry data demonstrated clear stripe patterns of γ-H2AX staining in the tumor tissue at 1 and 3 h after MPB irradiation, and these γ-H2AX-positive cells intermixed by 24 h (Fig. 6), which was comparable with the results reported by Crosbie *et al.* [9].

These facts may indicate that the influence of low dose rates (2–3 Gy/min) and beam divergence due to longer irradiation times (30–60 min) are not particularly significant provided that the total dose at the peak is the same in the mouse hind limb subcutaneous tumor model. Thus, low-flux X-rays can be considered suitable for the study of the biological effects of MPBs *in vivo*. Dose rates are 0.8–1.0 Gy/min for the current small linear accelerator installed with the CyberKnife® (Acuray, Sunnyvale, CA, USA). Although the energy value is different, our data with a low dose rate may be more practical and useful to the future development of MPB-based radiosurgery.

### MPBs with different kinds of peak and pitch widths ratios

Our study demonstrated that skin injury after MPB irradiation was milder and healed faster when compared with broad-beam irradiation at the equivalent doses for tumor control. Griffin *et al.* reported that 50/200 µm MPBs were more useful than those at 500/2000 µm, and the peak dose of 150 Gy was more efficient than 75 Gy [11]. In this study, we initially prepared 25/200 µm, 50/200 µm, 50/400 µm, 100/400 µm and 150/600 µm gold grid collimators; however, based on the peak dose and the peak and pitch widths ratios, we selected the 50/200 µm, 50/400 µm and 100/400 µm collimators for the study. Our data demonstrated that the suppression effect on tumor growth did not differ between these three MPBs at a peak dose of 100 Gy (Fig. 4); however, 100/400 µm and 50/200 µm MPBs were more effective than 50/400 µm MPB at 200 Gy, and 50/200 µm MPB tended to be more efficient than 100/400 µm MPB. These results are compatible with the reports by Uyama *et al.* and Griffin *et al.* revealing that narrow MPBs are more effective for tumor growth suppression than wide MPBs [11, 20]. Thus, we consider that 50/200 µm and 100/400 µm gold grid collimators are probably most suitable for the subcutaneous tumor model in mice.

### Biological response induced by MPB irradiation

Two main mechanisms have been postulated for biological effects of MPB irradiation [18]: blood vessel injury and the bystander effect. It has been reported that vessels in cancer tissue lack an intact integument layer, and cancer vessel endothelial cells are more radiosensitive than those in normal tissues [21, 22]. After MPB irradiation, these vulnerable endothelial cells are injured by a single fraction of high-dose X-rays at the irradiated area, inducing ischemia in both the irradiated and non-irradiated areas. Our data for CD34 staining demonstrated that the CD34-positive area was significantly reduced with both broad-beam and MPB irradiation over time, and the reduction rate of the positive area was greater for 100/400 µm MPBs than for 30 Gy broad beams. These findings may suggest that endothelial damage is not specific and is more prominent in MPB than in broad-beam irradiation. As for normal tissue damage, it has been reported that MPBs did not induce modification in either vessel diameter or density during the first 3 months after the exposure of mouse brains to 312 Gy MPBs [23]. Although MPBs might cause damage in endothelial cells in mouse brain, the damage might not be manifested as vascular damage in 3 months, because the lesions were limited to small areas, and endothelial cell death may result in different vascular changes in normal tissues. Collectively, MPB irradiation probably induces endothelial damage more prominently in tumor tissues than in normal tissues, which would be a major advantage of this method.

The other possible mechanism is the bystander effect. Our data demonstrated that γ-H2AX-positive cells intermixed over time with negative cells after MPB irradiation, particularly of 100/400 μm (Fig. 6). Cells irradiated with a peak dose of 200 Gy eventually die, and during the cell death process the dying and intact cells contact one another through this rapid intermixing phenomenon, which is then thought to promote direct cell-to-cell communication (the bystander effect). In specimens subjected to 30 Gy broad-beam irradiation, γ-H2AX positivity seemed lighter at 9 and 24 h when compared with those subjected to MPB irradiation, suggesting that the MPB-induced damage remains unrepaired for a longer period (Fig. 6). Furthermore, this may suggest the existence of an intermixing effect between lethally damaged and intact cells in the tumor tissue after MPB irradiation.

In addition, 10 Gy irradiation has been reported to induce bystander cell killing through both cell-to-cell contact and factors secreted from irradiated cells [24]. From these observations, it is reasonable to assume that bystander cell killing is a significant contributor to the overall antitumor effect of MPB irradiation. As for normal tissue, a bystander effect may also be involved in the cell repair following MPB irradiation. Dilmanian *et al.* irradiated *in vitro* endothelial cells and rat spinal cords with MPBs and concluded that the repair process could have involved beneficial bystander effects leading to tissue restoration [25]. These observations may indicate that certain MPB widths may induce different responses in normal and tumor tissues, as reported by Crosbie *et al.* [9]. Further investigation is warranted to clarify the various bystander effects in normal and tumor tissue following MPB irradiation that lead to cell rescue and cell killing, respectively. This should also include exploring for the optimal peak and pitch widths ratio.

### Future application of the system

High precision radiosurgical dose delivery of MPBs to solid tumors is the clinical goal of this research. This concept has significant potential for tumor control with low toxicity to normal tissue. With the limited depth reached by X-ray beams, this modality could also be applied to superficial tumors such as head-and-neck tumors. In addition, brain tumors may be suitable targets due the low risk of injury to normal brain tissue in animal models [17, 26]. When compact high-energy high-flux X-ray sources are developed, it may be possible to combine MPBs to facilitate the treatment of solid cancers that are located more deeply. Therefore, the biological effects of MPBs should be further clarified in both normal and tumor tissue to demonstrate their efficacy in cancer treatment.

In summary, low-flux X-ray MPBs were generated using a laboratory-scale X-ray generator. At equivalent doses for achieving the tumor control in a murine model, the resulting MPBs produced less toxicity to normal tissue when compared with conventional broad beams. Bystander effects and tumor vessel injury may be contributory mechanisms in the efficacy of MPBs; thus, further study is required.

## FUNDING

This work was conducted under a research contract between the University of Tsukuba and FUJIFILM Co. Ltd, and was partly supported by a Grant-in-Aid [24390287] from the Ministry of Education, Culture, Sports, Science and Technology of Japan. Funding to pay the Open Access publication charges for this article was provided by Grant of the University of Tsukuba.
